# Cord blood lipid mediator profiles associated with maternal passive smoking during pregnancy

**DOI:** 10.1016/j.toxrep.2026.102359

**Published:** 2026-10-17

**Authors:** Masanori Inoue, Nobuyuki Kawano, Shintaro Kishimoto, Tomoki Maeda, Kenji Ihara

**Affiliations:** Department of Pediatrics, Faculty of Medicine, Oita University, 1-1 Idaigaoka, Hasama, Yufu, Oita, 879-5593, Japan

**Keywords:** Passive smoking, Pregnancy, Umbilical cord blood, Lipid mediators, Oleoylethanolamide, Metabolomics

## Abstract

**Background:**

Maternal passive smoking (PS) during pregnancy remains a common environmental exposure associated with adverse perinatal outcomes. However, its effects on fetal lipid mediator profiles have not been fully characterized.

**Objective:**

To investigate whether maternal PS during pregnancy is associated with alterations in umbilical cord blood lipid mediator profiles in term neonates.

**Methods:**

We retrospectively analyzed umbilical cord blood samples from 56 term neonates, comprising 17 born to mothers with documented PS exposure and 39 born to mothers without PS exposure. Targeted lipid mediator profiling was performed using liquid chromatography–tandem mass spectrometry. Orthogonal partial least squares discriminant analysis, univariate analyses with Benjamini–Hochberg false discovery rate correction, receiver operating characteristic curve analysis, and exploratory multivariable linear regression were performed.

**Results:**

Four lipid mediators showed nominal differences between the groups, with higher levels of 14,15-LTE₄, platelet-activating factor, and 13-HpODE and lower levels of oleoylethanolamide (OEA) in the PS-exposed group. However, none remained statistically significant after correction for multiple comparisons. OEA was subsequently evaluated in post-hoc exploratory multivariable analysis and remained associated with maternal PS exposure after adjustment for maternal pre-pregnancy BMI and diabetes mellitus.

**Conclusions:**

This exploratory study identified several candidate cord blood lipid mediator signals related to maternal PS exposure, although none remained statistically significant after correction for multiple comparisons. These findings are hypothesis-generating but suggest that lipid mediator pathways involved in inflammatory and metabolic signaling may warrant further investigation in relation to prenatal tobacco smoke exposure.

## Highlights

• Maternal passive smoking was linked to candidate cord blood lipid mediator signals.
• Four lipid mediators showed nominal differences, but none survived FDR correction.
• OEA emerged as a candidate signal and was lower in exploratory adjusted analysis.
• Lipid mediator signaling may provide insight into fetal responses to tobacco smoke.

## Graphical Abstract

**Figure:**
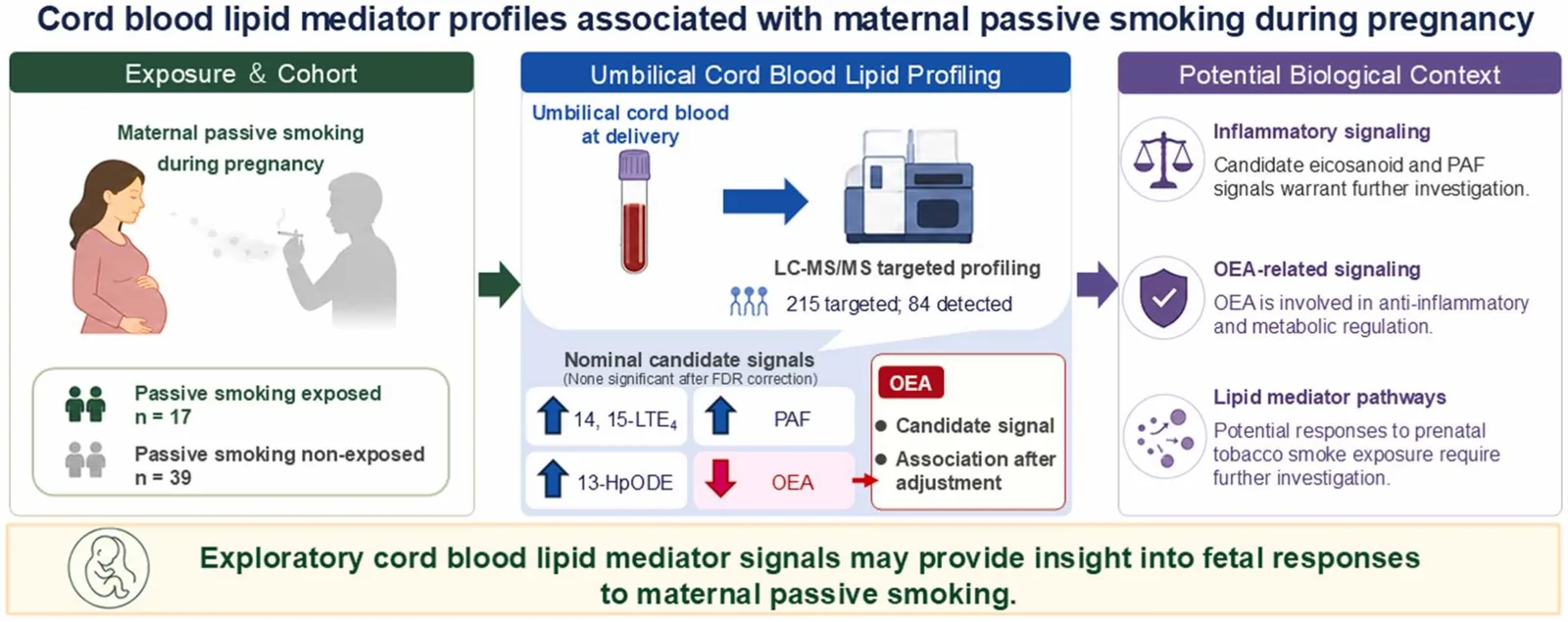

## 1 Introduction

Environmental tobacco smoke (ETS) and passive smoking (PS) remain major global health concerns. Despite declining smoking rates in many high-income countries, a substantial proportion of pregnant women worldwide are still involuntarily exposed to ETS in homes, workplaces, and public spaces. Recent meta-analyses have estimated that 30–60% of non-smoking pregnant women experience some degree of ETS exposure, with higher rates reported in low- and middle-income countries due to social norms regarding smoking and limited tobacco control measures [1], [2]. Such widespread exposure highlights the need to better understand its effects on fetal and neonatal health.

Maternal PS during pregnancy has been associated with adverse perinatal outcomes, such as fetal growth restriction, low birth weight, and preterm delivery [2], [3]. Maternal active smoking during pregnancy has stronger and more established links to placental dysfunction and compromised fetal lung development [4], [5]. After birth, infants exposed to ETS are at an increased risk of sudden infant death syndrome (SIDS), wheezing, and respiratory infections [6], [7]. Importantly, prenatal PS, which is more widespread and socially pervasive than active smoking, may predispose children to asthma, neurodevelopmental disorders, and metabolic dysfunction later in life.

One potential mechanism involves alterations in the intrauterine inflammatory and metabolic environment. Lipid mediators, including eicosanoids, platelet-activating factors, and endocannabinoid-related molecules, are key regulators of inflammation, vascular function, and immune development during fetal life [8]. Experimental studies have shown that tobacco smoke exposure can disrupt lipid signaling pathways and alter the expression of enzymes, receptors, and neurotrophic factors involved in inflammatory and developmental processes [6], [7], [9]. Such alterations may affect fetal programming and contribute to adverse health outcomes later in life. However, data regarding lipid mediator profiles in human neonates exposed to maternal passive smoking remain limited.

Umbilical cord blood provides a unique opportunity to directly evaluate the fetal biochemical environment at birth [8]. Comprehensive profiling of cord blood lipid mediators may provide insight into how prenatal environmental exposures influence inflammatory and metabolic signaling pathways during fetal development. Therefore, we performed targeted lipid mediator profiling of umbilical cord blood obtained from term neonates with and without maternal passive smoking exposure during pregnancy. We aimed to identify candidate lipid mediators associated with prenatal ETS exposure and to explore biological responses to maternal passive smoking during fetal development.

## 2 Methods

### 2.1 Study design and participants

This retrospective observational study was conducted at Oita University Hospital. We included term neonates (≥37 weeks of gestation) who were admitted to the neonatal intensive care unit (NICU) or growing care unit (GCU) between April 2020 and July 2024. Preterm infants were excluded to minimize the potential influence of prematurity on metabolic profiles. At our institution, asymptomatic term infants are typically managed by obstetricians, whereas neonates with clinical conditions such as respiratory distress, hypoglycemia, or fever, as well as those with birth weight < 2300 g or those classified as small for gestational age (birth weight and length below the 10th percentile), are admitted to the NICU or GCU for pediatric management. Neonates were excluded if they had major congenital anomalies, intrauterine infection, severe anemia due to fetomaternal transfusion syndrome, or maternal active smoking during pregnancy. Infants born to mothers receiving immunosuppressive therapy were also excluded. All participants were of Japanese ethnicity. The final study cohort comprised 56 neonates, representing 52 pregnancies. Both neonates were included from four twin pregnancies, whereas only one neonate was included from one additional twin pregnancy.

### 2.2 Assessment of maternal passive smoking exposure

Maternal PS exposure during pregnancy was routinely assessed for all mothers by midwives immediately before or after delivery as part of standard perinatal care. Mothers were directly asked about both their partner’s smoking status and whether they had been exposed to passive smoking during pregnancy, and their responses were documented in the midwifery records. For the present study, maternal PS exposure was categorized as present or absent based on the routinely recorded information regarding actual passive smoking exposure, rather than partner smoking status alone. Detailed information on exposure intensity, duration, timing, and type of tobacco product was not systematically collected. Biochemical validation of tobacco smoke exposure, such as cotinine measurement, was not performed. Therefore, although PS exposure was routinely assessed in all mothers using the same clinical procedure, exposure classification was based on maternal self-report.

### 2.3 Clinical data collection

Clinical data were obtained retrospectively from medical records. Neonatal variables included gestational age, birth weight, birth length, head circumference, sex, Apgar scores, and umbilical artery pH. Routine laboratory parameters obtained shortly after birth, including hematological, biochemical, inflammatory, and metabolic parameters, were also recorded. Biochemical and inflammatory parameters were measured using a LABOSPECT automated clinical chemistry analyzer (Hitachi High-Tech Corporation, Tokyo, Japan), and hematological parameters were measured using an XN-9000 automated hematology analyzer (Sysmex Corporation, Kobe, Japan).

Maternal variables included age, parity, body mass index before pregnancy and at delivery, obstetric complications, and medication use during pregnancy. Chorioamnionitis was diagnosed based on histopathological examination of the placenta. Maternal psychological status was assessed using the Edinburgh Postnatal Depression Scale (EPDS) at postpartum day 4 as part of routine clinical care.

### 2.4 Cord blood collection and processing

Umbilical venous blood samples were collected immediately after placental delivery. Samples were centrifuged for 10 min at 1200 × g within 3 h of collection, serum was aliquoted, and samples were stored at −80°C until analysis. No anticoagulants or enzyme inhibitors were added during sample processing. All samples underwent a single freeze–thaw cycle prior to liquid chromatography–tandem mass spectrometry (LC–MS/MS) measurement.

### 2.5 Lipid mediator profiling by LC–MS/MS

Umbilical cord blood serum samples (100 μL) were deproteinized with 1 mL 0.1% formic acid in methanol, vortexed for 30 s, gently shaken at 4 °C for 3 min, and centrifuged at 14,000 rpm for 10 min at 4 °C. The supernatant (1 mL) was diluted with 4 mL of 0.1% formic acid and subjected to solid-phase extraction using Strata-X cartridges (10 mg; Phenomenex, Torrance, CA, USA) on a vacuum manifold. The cartridges were conditioned with 1 mL 0.1% formic acid in methanol and 1 mL 0.1% formic acid in water, followed by sample loading (5 mL). After washing with 1 mL 0.1% formic acid and 1 mL 15% ethanol in 0.1% formic acid, lipid mediators were eluted with 300 μL 0.1% formic acid in methanol. The eluates were dried by vacuum centrifugation and reconstituted in 30 μL of 0.1% formic acid in methanol. A 20 μL aliquot of the reconstituted sample was subjected to LC–MS/MS analysis.

LC–MS/MS analyses were performed using an LCMS-8040 triple quadrupole mass spectrometer (Shimadzu, Kyoto, Japan) equipped with the Method Package for Lipid Mediators (ver. 3, Shimadzu). Samples were analyzed in multiple event monitoring mode. Lipid mediator abundance was assessed using the raw peak area of each mediator for exploratory profiling. Internal standards and calibration curves were not used, absolute concentrations were not determined, and compound-specific limits of detection (LOD) and quantification (LOQ) were not established. All 56 samples were analyzed within a single analytical run.

Peaks were initially identified using automated peak detection and subsequently visually inspected. When no peak was detected automatically and visual inspection confirmed the absence of a detectable peak, the value was recorded as zero. No imputation was performed for non-detects. Data acquisition and processing were performed using LabSolutions (Shimadzu).

### 2.6 Statistical analyses

Statistical analyses were performed using SPSS (version 29.0; IBM Corp., Armonk, NY, USA). Continuous variables were assessed for normality using the Shapiro–Wilk test and compared using Student’s *t*-test or the Mann–Whitney *U* test, as appropriate. Categorical variables were compared using the chi-square test or Fisher’s exact test, as appropriate. Fisher’s exact test was used when more than 20% of cells had expected counts less than 5.

Lipid mediator data were analyzed using MetaboAnalyst (version 6.0). The targeted LC–MS/MS panel included 215 lipid mediators, of which 84 were detected in at least one sample and included in the univariate analyses. All 84 detected lipid mediators were compared between the PS-exposed and PS-unexposed groups using the Mann–Whitney *U* test, and the resulting 84 p-values were adjusted for multiple comparisons using the Benjamini–Hochberg false discovery rate (FDR) procedure. For the principal unadjusted comparison of OEA between the PS-exposed and PS-unexposed groups, the between-group effect was additionally expressed as the Hodges–Lehmann estimate of the location shift with its 95% confidence interval. OPLS-DA was performed separately as an exploratory multivariate analysis, and lipid mediators with variable importance in projection (VIP) scores > 1.0 were identified. The VIP criterion was not used to select or restrict the lipid mediators included in the univariate analyses or FDR correction.

Detection frequencies were calculated for each of the 84 lipid mediators in the overall cohort and separately for the PS-exposed and PS-unexposed groups. As a sensitivity analysis, the univariate comparisons were repeated after restricting the analysis to lipid mediators detected in at least 80% of the study samples, consistent with the 80% rule commonly used to reduce the influence of variables with excessive missing values in metabolomics analyses [10], [11]. The resulting p-values were adjusted for multiple comparisons across the mediators included in the sensitivity analysis using the Benjamini–Hochberg FDR procedure.

As an exploratory analysis, receiver operating characteristic (ROC) curves were generated for the four lipid mediators that showed nominal between-group differences (raw p < 0.05) to describe their ability to distinguish PS-exposed from PS-unexposed participants within the present dataset. Among the four candidate lipid mediators, OEA had the highest AUC in exploratory ROC analysis and was therefore selected for post-hoc exploratory multivariable linear regression analysis. Because OEA was selected based on analyses performed in the same dataset, the regression analysis was considered hypothesis-generating rather than confirmatory. Given the exploratory nature and limited sample size of the study, the multivariable model was restricted to a small number of covariates selected based on biological and temporal considerations rather than between-group statistical significance. Maternal pre-pregnancy BMI and maternal diabetes mellitus (including gestational and pre-gestational diabetes mellitus) were included as potential maternal metabolic confounders because of their plausible relationships with maternal and fetal lipid metabolism [12], [13], [14].

Because both neonates from four twin pregnancies were included in the primary analysis, a sensitivity analysis was performed using one neonate per pregnancy to account for potential within-pregnancy clustering. For each twin pair in which both neonates were included, the first-born neonate was retained, resulting in 52 neonates from 52 independent pregnancies. The OEA-related analyses were repeated using this dataset.

Statistical significance was defined as a two-sided p < 0.05.

### 2.7 Ethical approval

This study was performed in accordance with the Declaration of Helsinki and the Ethical Guidelines for Medical and Biological Research Involving Human Subjects formulated by the Japanese government. This study was reviewed and approved by the Ethics Committee of Oita University Faculty of Medicine (Approval No. 3147, June 2025). Informed written consent was substituted by the informed opt-out procedure because of the retrospective nature of the study using existing clinical data and stored umbilical cord blood samples. Information regarding this study was made publicly available on the website of Oita University Hospital to give participants the opportunity to opt out, and those who did not opt out were considered to have provided tacit consent for study participation. Anonymous clinical data and samples were used for the analysis, and individuals cannot be identified based on the data presented. The waived written consent and the informed opt-out procedure were approved by the Ethics Committee of Oita University Faculty of Medicine.

## 3 Results

### 3.1 Perinatal characteristics

A total of 56 term neonates were included in this study, comprising 17 infants born to mothers with passive smoking exposure and 39 born to unexposed mothers (Fig. 1). The clinical characteristics of the neonates and their mothers are summarized in Table 1. No significant differences were observed between the groups in gestational age, birth weight, birth length, head circumference, or routine laboratory parameters obtained shortly after birth. The 1-minute Apgar score was slightly higher in the passive smoking–exposed group than in the unexposed group (median [interquartile range, IQR]: 8 [7], [8] vs. 7 [5], [6], [7], [8], p = 0.021). However, no significant differences were observed in the 5-minute Apgar score or umbilical artery pH. Maternal and neonatal background characteristics were otherwise comparable between the groups. The duration of sample storage did not differ significantly between the PS-exposed and PS-unexposed groups (median [IQR], 1114 [904–1490] vs 1093 [843–1442] days, respectively; p = 0.776).

**Fig. 1:**
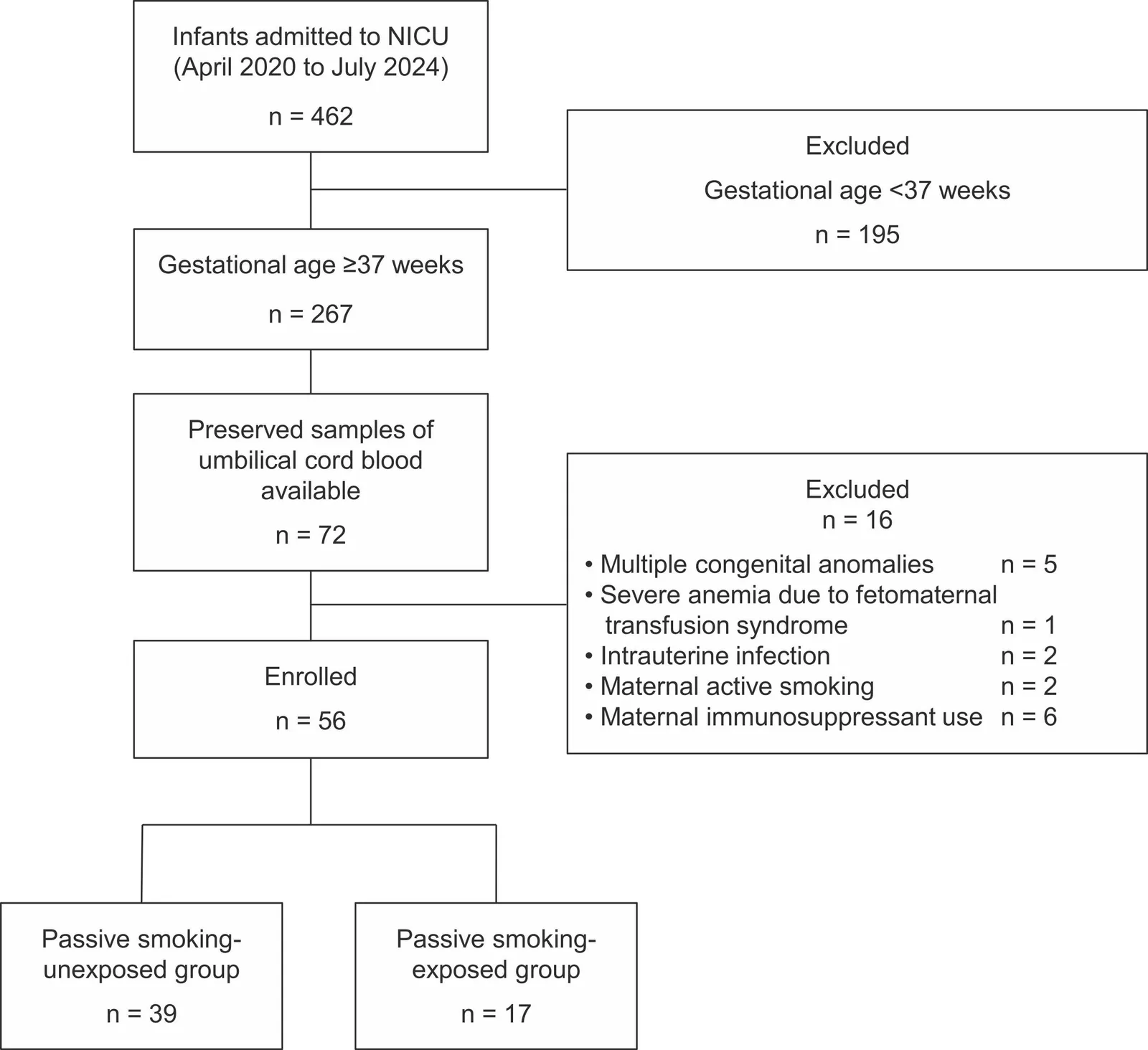
Flow diagram of participant selection and exclusion criteria.

**Table 1:** Perinatal characteristics of term infants with maternal passive smoking exposure.

	PS-unexposed group n = 39	PS-exposed group n = 17	*p*
Characteristics of infants			
Male, n (%)	21 (54)	9 (53)	0.950
Gestational age, median (IQR), weeks	37.8 (37.1–39.1)	37.9 (37.4–38.9)	0.688
Birth weight, median (IQR), g	2857 (2425–3304)	2685 (2418–3267)	0.755
Birth length, mean (SD), cm	48.5 (2.6)	48.3 (2.2)	0.829
Birth head circumference, mean (SD), cm	33.7 (1.6)	33.7 (1.6)	0.972
Twin, n (%)	7 (18)	2 (12)	0.707
Apgar score 1 min, median (IQR)	7 (5−8)	8 (7−8)	0.021*
Apgar score 5 min, median (IQR)	8 (8−9)	9 (8−9)	0.135
Umbilical artery pH, median (IQR)	7.28 (7.25–7.31)	7.28 (7.23–7.30)	0.428
Chorioamnionitis, n (%)	6 (15)	3 (18)	1.000
Neonatal laboratory data immediately after birth			
Serum TP level, mean (SD), g/dL	5.5 (0.6)	5.5 (0.5)	0.944
Serum ALB level, mean (SD), g/dL	3.5 (0.3)	3.5 (0.3)	0.981
Serum T-Bil level, median (IQR), mg/dL	2.0 (1.9–2.5)	2.2 (1.7–2.5)	0.882
Serum AST level, median (IQR), IU/L	30.0 (22.6–39.3)	28.6 (23.0–32.3)	0.402
Serum ALT level, median (IQR), IU/L	5.3 (4.4–9.5)	6.7 (4.4–7.8)	0.434
Serum ALP level, median (IQR), IU/L	197 (168−239)	193 (179−271)	0.551
Serum CK level, mean (SD), IU/L	339 (126)	329 (115)	0.786
Serum LDH level, mean (SD), IU/L	384 (84)	350 (62)	0.148
Serum T-chol level, mean (SD), IU/L	76.2 (19.0)	72.8 (20.5)	0.558
Serum BUN level, mean (SD), mg/dL	9.0 (2.5)	8.4 (1.8)	0.417
Serum CRE level, median (IQR), mg/dL	0.66 (0.58–0.79)	0.66 (0.60–0.71)	0.402
Serum UA level, mean (SD), mg/dL	5.7 (1.7)	5.0 (1.0)	0.162
Serum Na level, mean (SD), mEq/L	138 (2)	139 (2)	0.635
Serum K level, mean (SD), mEq/L	4.7 (0.5)	4.8 (0.7)	0.659
Serum Cl level, mean (SD), mEq/L	107 (3)	108 (2)	0.300
Serum Ca level, median (IQR), mg/dl	9.7 (9.4–10.2)	9.8 (9.5–10.3)	0.589
Serum P level, mean (SD), mg/dL	5.7 (0.9)	5.6 (0.6)	0.521
Serum CRP level, mean (IQR), mg/dL	0.00 (0.00–0.01)	0.00 (0.00–0.01)	0.773
WBC count, median (IQR), /L	13,365 (11,450−17,050)	13,540 (11,595−16,055)	0.841
Hb level, median (IQR), g/dL	16.2 (15.3–17.6)	16.8 (15.8–18.3)	0.325
PLT count, median (IQR), × 10^3^/L	298 (243−334)	264 (249−337)	0.542
Serum IgG level, median (IQR), mg/dL	929 (846–1179)	1020 (860–1190)	0.475
Serum IgM level, mean (SD), mg/dL	10 (4)	10 (3)	0.798
Blood glucose level, median (IQR), mg/dL	51 (40−69)	62 (52−81)	0.118
Characteristics of mothers			
Maternal age, mean (SD), years old	34 (5)	32 (6)	0.367
Maternal weight at delivery, mean (SD), kg	64.4 (14.4)	66.9 (12.3)	0.533
Maternal pre-pregnancy weight, median (IQR), kg	53.3 (46.3–70.8)	57.9 (48.8–70.8)	0.369
Maternal height, median (IQR), cm	158.1 (154.1–161.1)	159.1 (153.4–162.9)	0.599
Maternal BMI at delivery, mean (SD), kg/m^2^	25.8 (5.3)	26.6 (4.3)	0.595
Maternal pre-pregnancy BMI, median (IQR), kg/m^2^	22.0 (19.5–28.3)	23.1 (19.8–28.3)	0.374
Nulliparity, n (%)	13 (33)	8 (47)	0.329
Caesarean delivery, n (%)	31 (79)	14 (82)	1.000
PROM, n (%)	2 (5)	3 (18)	0.158
Alcohol use during pregnancy, n (%)	0 (0)	1 (6)	0.304
Gestational hypertension, n (%)	3 (8)	2 (12)	0.634
Diabetes mellitus, n (%)	2 (5)	4 (24)	0.062
Collagen disease, n (%)	0 (0)	1 (6)	0.304
Perinatal sedation-related factors, n (%)	10 (26)	2 (12)	0.418
EPDS score, median (IQR)	3 (1−5)	2 (1−6)	0.857

PS, passive smoking; TP, total protein; ALB, albumin; T-Bil, total bilirubin; AST, aspartate aminotransferase; ALT, alanine aminotransferase; ALP, alkaline phosphatase; CK, creatine kinase; LDH, lactate dehydrogenase; T-chol, total cholesterol; BUN, blood urea nitrogen; CRE, creatinine; UA, uric acid; Na, sodium; K, potassium; Cl, chloride; Ca, calcium; P, phosphorus; CRP, C-reactive protein; WBC, white blood cell; Hb, hemoglobin; PLT, platelet; IgG, immunoglobulin G; IgM, immunoglobulin M; BMI, body mass index; PROM, premature rupture of membranes; Diabetes mellitus, includes gestational and pre-gestational diabetes mellitus; Perinatal sedation-related factors, defined as general anesthesia during cesarean section or maternal use of psychotropic medications (e.g., benzodiazepines, antipsychotics, or anxiolytics) during pregnancy that may affect the neonate; EPDS, Edinburgh postnatal depression scale assessed at postpartum day 4; IQR, interquartile range; SD, standard deviation.

### 3.2 Multivariate analysis of lipid mediator profiles

OPLS-DA was performed as an exploratory analysis of the overall lipid mediator profile (Fig. 2). Although the score plot showed visual separation between the PS-exposed and PS-unexposed groups, the model yielded an R²Y of 0.710 but showed minimal predictive ability (Q² = 0.0043). Permutation testing (n = 100) did not support statistically significant model performance (empirical p = 0.41 for R²Y and p = 0.07 for Q²). Therefore, the OPLS-DA findings were not considered evidence of reliable discrimination between the groups.

**Fig. 2:**
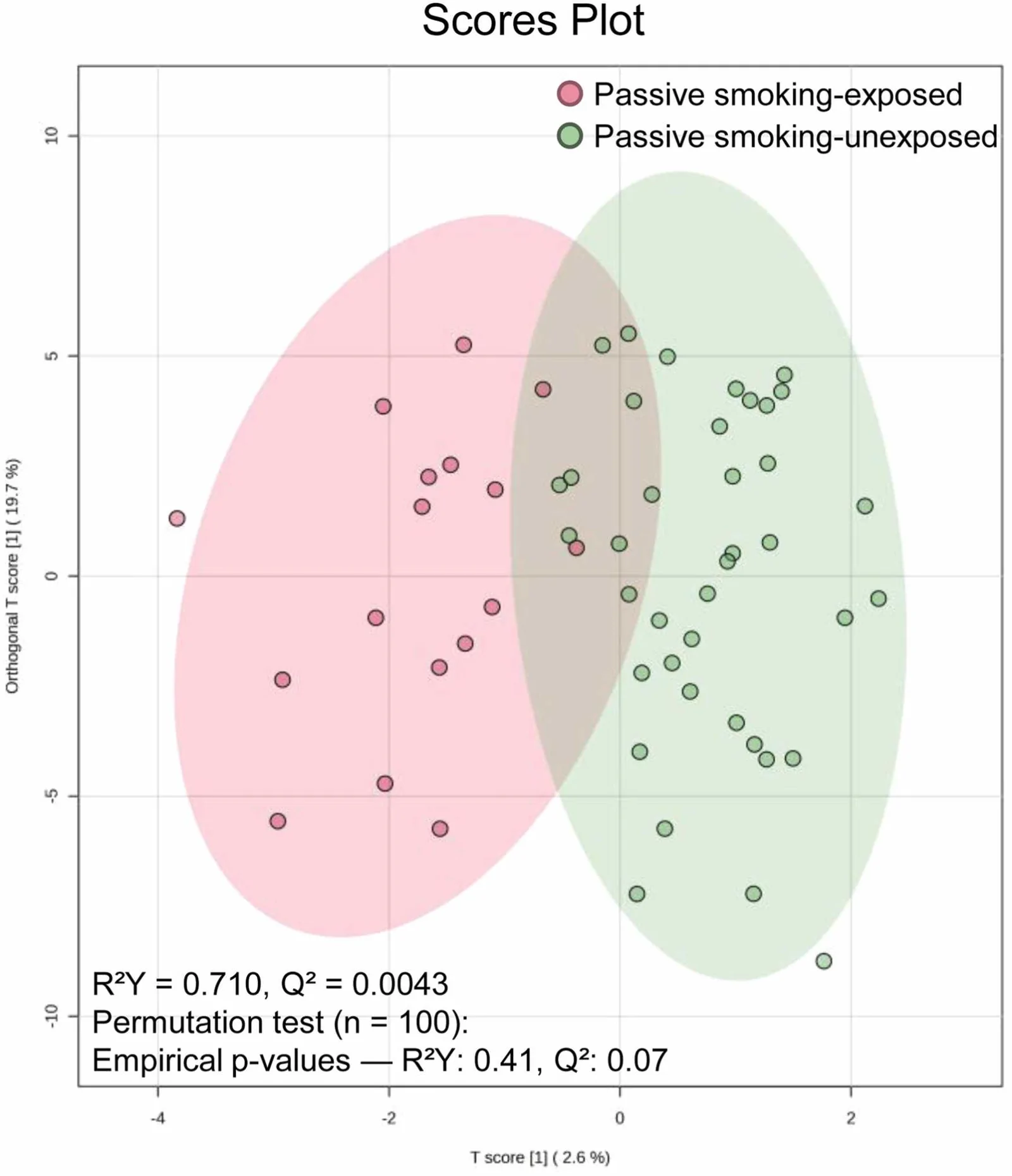
OPLS-DA score plot of lipid mediators in cord blood by maternal passive smoking status. OPLS-DA score plot of the passive smoking-exposed and passive smoking-unexposed groups based on cord blood lipid mediator profiles. Model parameters were R²Y = 0.710 and Q² = 0.0043. Permutation testing (n = 100) yielded empirical p-values of 0.41 for R²Y and 0.07 for Q². The analysis was performed using MetaboAnalyst 6.0.

### 3.3 Identification of differential lipid mediators

To identify lipid mediators contributing to group separation, combined multivariate and univariate analyses were performed. Among the 28 candidate lipid mediators with VIP scores > 1.0 identified by OPLS-DA (Fig. 3A and Supplementary Table S2), four showed nominal differences between the groups in the Mann–Whitney *U* test (Fig. 3B and Table 2). Compared with the PS-unexposed group, levels of 14,15-LTE₄ (log₂FC = 1.28, raw p = 0.002), platelet-activating factor (PAF; log₂FC = 1.16, raw p = 0.031), and 13-HpODE (log₂FC = 0.92, raw p = 0.048) were higher in the PS-exposed group, whereas OEA was lower (log₂FC = −0.42, raw p = 0.016). For OEA, the Hodges–Lehmann estimate of the location shift (PS-exposed minus PS-unexposed) was −2821 (95% CI, −5023 to −711). However, none of these lipid mediators remained statistically significant after Benjamini–Hochberg correction for multiple comparisons (Table 2).

**Fig. 3:**
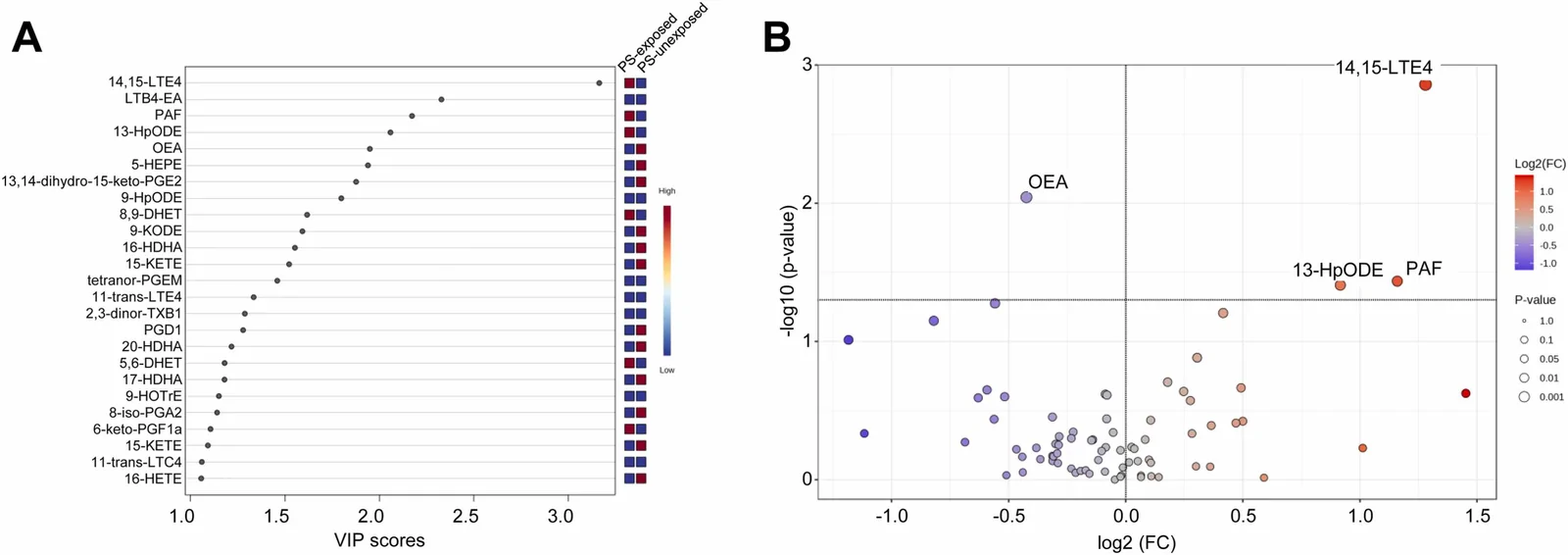
Candidate lipid mediators associated with maternal passive smoking exposure. (A) Variable Importance in Projection (VIP) scores from orthogonal partial least squares discriminant analysis (OPLS-DA) for 28 lipid mediators. Lipid mediators with VIP scores > 1.0 in the exploratory OPLS-DA model are shown. (B) Volcano plot showing the distribution of lipid mediators based on log₂ fold change (FC) and –log₁₀ (p-value). Metabolites with nominal p values < 0.05 are highlighted. Analyses were performed using MetaboAnalyst 6.0.

**Table 2:** Exploratory and multivariable analyses of lipid mediators associated with maternal passive smoking exposure.

A. Candidate lipid mediators identified in exploratory analyses						
Lipid mediator	VIP	log₂(FC)		Raw p-value		FDR (BH)
14,15-LTE4	3.16	1.28		0.002		0.168
OEA	1.95	-0.42		0.016		0.672
PAF	2.17	1.16		0.031		0.708
13-HpODE	2.06	0.92		0.048		0.708
B. Linear regression analyses of ln-transformed cord blood OEA peak area						
Variable	B		95% CI		p-value	
Maternal PS exposure	−0.329		−0.576 to −0.083		0.010	
Maternal pre-pregnancy BMI	0.003		−0.019–0.024		0.798	
Maternal diabetes mellitus	0.019		−0.354–0.392		0.918	

Panel A: VIP, variable importance in projection derived from the OPLS-DA model; VIP > 1 was considered indicative of important variables. log₂(FC), log₂ fold change calculated from raw peak areas. Raw p-values were obtained using the Mann–Whitney *U* test. False discovery rate (FDR) was calculated using the Benjamini–Hochberg procedure.

Panel B: The dependent variable was the natural logarithm-transformed raw peak area of cord blood OEA. Maternal diabetes mellitus included gestational and pre-gestational diabetes mellitus. The analysis included 55 participants because maternal pre-pregnancy BMI was unavailable for one participant. BMI, body mass index; LTE₄, leukotriene E₄; OEA, oleoylethanolamide; PAF, platelet-activating factor; HpODE, hydroperoxy-octadecadienoic acid.

Detection frequencies of the 84 lipid mediators included in the original univariate analyses are shown in Supplementary Table S3. Forty-six lipid mediators were detected in at least 80% of the samples and were included in the sensitivity analysis. Among these, OEA, which was detected in all 56 samples, showed the lowest raw p-value (p = 0.016), but did not remain statistically significant after FDR correction (FDR = 0.743). The other three candidate mediators, 14,15-LTE₄, PAF, and 13-HpODE, were detected in only 4 (7.1%), 2 (3.6%), and 4 (7.1%) samples, respectively, and were therefore excluded from the sensitivity analysis.

### 3.4 Exploratory ROC and multivariable analyses of candidate lipid mediators

ROC curves were explored for the four candidate lipid mediators that showed nominal between-group differences (Fig. 4). OEA had an AUC of 0.704 (95% CI: 0.558–0.851), whereas the AUC values for 14,15-LTE₄, platelet-activating factor, and 13-HpODE ranged from 0.558 to 0.618. These estimates were derived from the same dataset used to identify the candidate mediators and were not internally or externally validated.

**Fig. 4:**
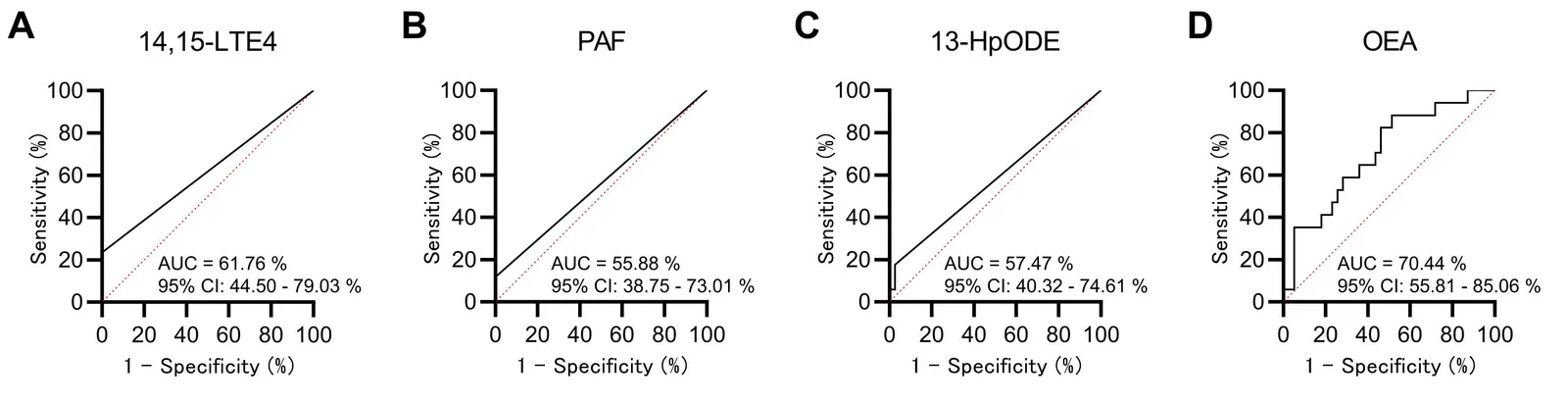
Receiver operating characteristic (ROC) curves of candidate lipid mediators associated with maternal passive smoking exposure. ROC curves for (A) 14,15-LTE₄, (B) platelet-activating factor (PAF), (C) 13-HpODE, and (D) oleoylethanolamide (OEA) were generated to explore their ability to distinguish PS-exposed from PS-unexposed participants within the present dataset. Graphs and statistical analyses were performed using GraphPad Prism 8 J.

Based on this exploratory analysis, OEA was subsequently evaluated in post-hoc exploratory multivariable linear regression analysis. Because OEA was selected based on analyses performed in the same dataset, the regression analysis was considered hypothesis-generating rather than confirmatory. After adjustment for maternal pre-pregnancy BMI and maternal diabetes mellitus, maternal passive smoking exposure remained associated with lower cord blood OEA peak area (B = −0.329, 95% CI: −0.576 to −0.083, p = 0.010) (Table 2).

In a sensitivity analysis restricted to one neonate per pregnancy (n = 52), OEA remained nominally lower in the PS-exposed group (Mann–Whitney *U* test, p = 0.030), with a similar AUC of 0.691. In the post-hoc exploratory multivariable analysis (n = 51), maternal passive smoking exposure remained associated with lower cord blood OEA peak area after adjustment for maternal pre-pregnancy BMI and maternal diabetes mellitus (B = −0.309, 95% CI: −0.547 to −0.070, p = 0.012).

## 4 Discussion

In this exploratory study, four cord blood lipid mediators showed nominal differences between neonates with and without maternal passive smoking exposure; however, none remained statistically significant after correction for multiple comparisons. Specifically, 14,15-LTE₄, platelet-activating factor (PAF), and 13-HpODE tended to be higher, whereas OEA tended to be lower in the exposed group. Among these candidate mediators, OEA had the highest AUC in exploratory ROC analysis and was subsequently evaluated in post-hoc exploratory multivariable analyses, in which lower OEA levels were associated with maternal passive smoking exposure after adjustment for maternal pre-pregnancy BMI and maternal diabetes mellitus. Taken together, these findings should be considered hypothesis-generating and suggest candidate lipid mediator signals for future investigation rather than demonstrating established alterations in cord blood lipid mediator profiles.

Previous metabolomic studies support the possibility that prenatal tobacco smoke exposure is associated with alterations in neonatal lipid metabolism, although the specific metabolites identified have varied across studies. Rolle-Kampczyk et al. reported differences in several phosphatidylcholines and sphingomyelin in cord blood according to maternal smoking status [15], while a larger population-based study identified lower concentrations of several phosphatidylcholine and lysophosphatidylcholine species in neonates exposed to maternal smoking during pregnancy [16]. More recently, newborn metabolomic profiling using objective maternal tobacco exposure biomarkers identified metabolic perturbations associated with prenatal tobacco exposure and also implicated lipid-related pathways, including leukotriene and arachidonic acid metabolism, in early birth outcomes [17]. In addition, maternal passive smoking has previously been associated with increased oxidative stress in cord blood [18]. Although these studies differ from the present study in exposure assessment, study population, biospecimen, and metabolomic platform, collectively they provide a broader context for our exploratory lipid mediator signals. Importantly, the specific candidate mediators identified in our study, including OEA, have not been consistently reported in these previous neonatal studies, and none of our between-group differences remained significant after FDR correction. Thus, our findings identify candidate lipid mediator signals for further investigation rather than confirming previously established metabolite changes.

Among the candidate lipid mediators identified in the present study, 14,15-LTE₄, PAF, and 13-HpODE are all linked to inflammatory signaling pathways. 14,15-LTE₄, also known as eoxin E₄, is a proinflammatory eicosanoid generated through the 15-lipoxygenase pathway and is upregulated in response to inflammatory stimuli [19]. Elevated levels have been reported in pediatric airway inflammatory diseases, including asthma and bronchial hyper-responsiveness [20]. PAF promotes leukocyte migration and vascular inflammation and has been shown to accumulate in response to cigarette smoke exposure [21], [22]. 13-HpODE is an oxidized linoleic acid metabolite associated with oxidative stress and inflammatory responses [23]. Previous studies have shown that tobacco smoke exposure can disrupt lipid signaling pathways and promote inflammatory responses. However, 14,15-LTE₄, PAF, and 13-HpODE were detected in only a small proportion of samples in the present study and were excluded from the sensitivity analysis restricted to adequately detected mediators. Therefore, these nominal differences should be interpreted cautiously as exploratory signals rather than evidence of inflammatory alterations in the fetal environment.

Among the four candidate lipid mediators, OEA was the only mediator with lower levels in the passive smoking–exposed group. OEA is a fatty acid ethanolamide derived from oleic acid and has been implicated in anti-inflammatory, antioxidant, and metabolic regulation, including signaling through peroxisome proliferator-activated receptor-α (PPAR-α) [24], [25], [26], [27], [28]. Experimental studies have also suggested that cigarette smoke exposure may affect endocannabinoid-related lipid signaling, including pathways involved in OEA synthesis and degradation [29], [30]. These previous findings provide a possible biological context for the lower OEA signal observed in the present study. Although OEA did not remain statistically significant after correction for multiple comparisons and its downstream pathways were not assessed, the consistent direction of the OEA signal across the exploratory analyses suggests that OEA may warrant further investigation as a candidate lipid mediator associated with prenatal passive smoking exposure.

The finding that the 1-minute Apgar score was lower in the unexposed group appears counterintuitive. However, no corresponding differences were observed in the 5-minute Apgar score or umbilical artery pH, suggesting that this finding is unlikely to reflect clinically relevant neonatal compromise. Potential unmeasured factors, such as obstetric anesthesia or maternal medications, may also have contributed to this unexpected finding [31], [32]. In addition, the inclusion of only NICU- or GCU-admitted neonates may have introduced selection bias, as infants with lower Apgar scores are more likely to be hospitalized.

Several limitations should be considered when interpreting the present findings. First, although several candidate lipid mediators showed nominal differences between groups, none remained statistically significant after correction for multiple comparisons. In addition, OEA was selected for multivariable analysis based on nominal between-group differences and exploratory ROC analysis performed in the same dataset. This data-driven selection introduces the potential for post-selection inference; therefore, the resulting regression estimates and p-values should be interpreted as exploratory and hypothesis-generating rather than confirmatory. Similarly, the ROC estimates were derived from the same small dataset used to identify the candidate lipid mediators and were not internally or externally validated. Therefore, they may be optimistic and should not be interpreted as evidence that OEA is a validated biomarker of passive smoking exposure. Second, the sample size was relatively small, particularly in the exposed group, limiting statistical power. In addition, the study population consisted of term neonates admitted to the NICU or GCU rather than an unselected population of healthy term neonates. Moreover, preserved cord blood samples were available for only 72 of the 267 potentially eligible neonates before further exclusions. This selection process may have introduced selection bias and limits the generalizability of our findings beyond this selected clinical cohort. Third, maternal passive smoking exposure was routinely assessed in all mothers by direct questioning as part of standard perinatal care; however, exposure classification relied on maternal self-report without biochemical validation. The absence of quantitative exposure data, including exposure intensity, duration, timing, and cotinine measurements, may have resulted in exposure misclassification. Comparison with an objective measure of tobacco smoke exposure, such as cotinine, would also be required to evaluate the potential utility of OEA as an exposure biomarker. Fourth, although multivariable analyses were performed for OEA, potential confounding factors were not comprehensively addressed in the lipid profiling analysis. Maternal clinical characteristics, dietary factors, environmental exposures, and other lifestyle variables may have influenced lipid mediator profiles. Finally, lipid mediator abundance was assessed using raw LC–MS/MS peak areas without internal-standard normalization or calibration curves, and absolute concentrations were not determined. In addition, pooled QC samples were not included, and analytical precision, extraction recovery, matrix effects, and instrument drift were not formally evaluated. Serum was collected without specific inhibitors; therefore, ex vivo generation or degradation of some lipid mediators during clotting and sample processing cannot be excluded. These pre-analytical and analytical limitations should be considered when interpreting the exploratory lipid mediator findings.

In summary, this exploratory study identified several candidate cord blood lipid mediator signals in relation to maternal passive smoking exposure in a selected clinical cohort of term neonates admitted to the NICU or GCU, although none remained statistically significant after correction for multiple comparisons. The findings, including the exploratory observations for OEA, should be considered hypothesis-generating and require validation in larger prospective cohorts with objective assessment of tobacco smoke exposure.

## CRediT authorship contribution statement

**Masanori Inoue:** Writing – original draft, Investigation, Formal analysis, Data curation, Conceptualization. **Nobuyuki Kawano:** Investigation, Data curation, Conceptualization. **Shintaro Kishimoto:** Methodology, Investigation. **Tomoki Maeda:** Formal analysis. **Kenji Ihara:** Writing – review & editing, Supervision.

## Funding

This research did not receive any specific grant from funding agencies in the public, commercial, or not-for-profit sectors.

## Declaration of Competing Interest

The authors declare that they have no known competing financial interests or personal relationships that could have appeared to influence the work reported in this paper.

## Data Availability

Data will be made available on request.
